# Influence of Severe Carotid Stenosis on Cognition, Depressive Symptoms and Quality of Life

**DOI:** 10.2174/1745017901713010168

**Published:** 2017-10-19

**Authors:** Elina Pucite, Ildze Krievina, Evija Miglane, Renars Erts, Dainis Krievins

**Affiliations:** 1 Department of Neurology, Pauls Stradins Clinical University Hospital, , Latvia; 2 Department of Neurology and Neurosurgery, Riga Stradins University, Riga, Latvia; 3 Department of Physics, Riga Stradins University, , Latvia; 4 Vascular Surgery Centre, Pauls Stradins Clinical University Hospital, , Latvia; 5 Department of Surgery, University of Latvia, Faculty of Medicine, , Latvia

**Keywords:** Carotid stenosis, Cognitive impairment, Depressive symptoms, Quality of life, Ischemic stroke, Low health

## Abstract

**Background:**

Carotid artery disease is not just a causal risk factor of ischemic stroke, but may predispose patients to depressive symptoms and low health related quality of life (HRQoL).

**Objectives:**

The objectives of the present study were to assess the association between severe carotid artery stenosis (CAS) and cognitive impairment, frequency of depressive symptoms and status of HRQoL.

**Methods:**

Cross - sectional study involved 55 patients with severe CAS and 54 patients with lower extremity peripheral artery disease (PAD). Cognitive impairment was assessed using Montreal Cognitive Assessment Scale (MoCA), depressive symptoms - PHQ-9 scale. HRQoL was measured using Medical Outcome Survey Short Form version 2 (SF-36v2).

**Results:**

Median MoCA score 24 [23;26] was significantly lower in patients with severe CAS than in patients with PAD - 26 [25-28],(*p*=0.005; effect size *r*=0.3). There was no statistically significant difference of median PHQ-9 scores the in CAS group (median PHQ-9 score 4.0 [5]) and in the PAD group (median PHQ-9 score 5.5 [7]), (*p*=0.08, effect size *r*=0.18). Mean SF-36v2 scores were similar in CAS and PAD groups except for bodily pain (*p*=0.001, Cohen's *d* value = 0.77) and vitality (*p*=0.02, Cohen's *d* value = 0.49).

**Conclusion:**

In summary, our findings indicate that severe CAS could play a role in cognitive decline. Further studies should be conducted using larger patient cohorts without ischemic brain lesions and with balanced vascular risk profiles to investigate impact of CAS on cognition. There was no association between severe CAS and depressive symptoms in the present study. As patients with severe CAS did not exhibit physical symptoms, HRQoL was better for those patients than for patients with lower extremity PAD.

## 1 INTRODUCTION

Atherosclerosis is a chronic systemic inflammatory disease affecting all arteries in the body [1]. Age-adjusted atherosclerotic cardiovascular disease (ACD) mortality rate trends have decreased globally, but the absolute number of ACD deaths is increasing in part due to the growth and the aging of the population, making atherosclerosis a leading cause of mortality world-wide [2]. The widespread occurrence of the atherosclerosis demands a closer look at how it affects quality of life.

Carotid artery atherosclerosis is not just a causal risk factor of ischemic stroke [3, 4], it also appears to be an independent risk factor for cognitive impairment [5] and may predispose patients to depressive symptoms and low health related quality of life (HRQoL) [6]. The assessment of cognitive performance has been reported in patients with severe carotid artery stenosis (CAS) [7], but the results do not show clear interdependency. It remains controversial [8], whether patients with asymptomatic severe CAS suffer from cognitive impairment.

A relevant factor associated with atherosclerosis is depression. High prevalence of depression in cardiovascular disease [9] and stroke patients [10] is well documented in literature. Considering reports of cognitive performance in patients with asymptomatic carotid stenosis due to hypoperfusion, microemboli, altered cerebrovascular reactivity and impaired regional functional connectivity [7], these pathogenetic mechanisms could theoretically also favour depressive symptoms besides the current concept of “vascular depression” [11]. Therefore if CAS itself would promote cognitive impairment and depressive symptoms, revascularisation may prevent not only cognitive decline and depression but also improve quality of life.

The objectives of the present study were to assess the association between severe CAS and cognitive impairment, frequency of depressive symptoms and status of HRQoL. We hypothesised that patients with severe carotid stenosis would have worse cognitive performance, depressive symptoms and HRQoL than patients with lower extremity peripheral artery disease (PAD).

## 2 MATERIALS AND METHODS

### 2.1 Design and Study Population

The study involved 55 patients undergoing carotid artery endarterectomy (CEA) for clinically severe carotid stenosis (≥ 70% luminal narrowing) and 54 patients with lower extremity PAD undergoing iliac or femoral artery revascularisation as a control group at the Pauls Stradins Clinical University Hospital from March 2016 to February 2017.

All consenting patients with severe CAS enrolled in this prospective exploratory study met the following inclusion criteria: age 18 years or older, severe extracranial CAS ≥ 70% and lower extremity PAD. CAS was estimated with computed tomography angiography and defined according to North American Symptomatic Carotid Artery Endarterectomy Trial (NASCET) criteria [12]; symptomatic carotid stenosis was considered if a minor stroke (National Institute of Health Stroke Scale (NIHSS) <4, modified Rankin Scale 0 – 2 at the time of inclusion), transient ischemic attack (TIA) or *Amaurosis fugax* occurred within 6 months; asymptomatic carotid stenosis was considered if Asymptomatic Carotid Atherosclerosis Study criteria were met [13]. Lower extremity PAD was defined if it was documented in previous medical reports or the patient had symptoms of claudication. Exclusion criteria were major stroke (modified Rankin Scale III-V), patients with antidepressant therapy and progressive cerebral disease (tumour, multiple sclerosis).

Fifty four consenting patients with severe PAD undergoing iliac or femoral artery revascularisation served as control patients to match patients on demographic and cardiovascular risk factor variables. Severe PAD was defined as lifestyle - limiting claudication with inadequate response to guideline - directed management and therapy [14]. All control group patients underwent duplex ultrasound assessment of the carotid arteries and were included if CAS was <50%. Control patients were excluded based on history of stroke, TIA or carotid artery revascularisation, antidepressant therapy and progressive cerebral disease (tumour, multiple sclerosis).

### 2.2 Basic Characteristics

All patients were assessed 1-3 days before surgical management by a trained neurologist in a quiet and ambient room at the hospital. Basic demographic characteristics (age, sex, education), anthropometric and lifestyle characteristics (weight, height, smoking), data on comorbidities (history of stroke or TIA, coronary artery disease, arterial hypertension (AH), chronic heart failure, atrial fibrillation, diabetes mellitus, PAD), use of medications and neurological examination were recorded on standardized form. Body mass index (BMI) was calculated as weight (kg) divided by the square of high in metres (m^2^). Participants were classified as cigarette smokers if they were current smokers or had quit smoking within 5 years before enrolment. History of TIA or minor stroke was collected from previous medical records. Coronary artery disease was defined as a previous diagnosis of angina pectoris, myocardial infarction. AH was defined as values ≥ 140mmHg systolic blood pressure and/or ≥90mmHg diastolic blood pressure or current treatment with antihypertensive drugs [15]. Patients who had New York Heart Association (NYHA) class II to III chronic heart failure were included [16]. Atrial fibrillation was defined as documented diagnosis of all patterns of atrial fibrillation according to European Society of Cardiology guidelines [17]. Diabetes mellitus was defined as a previous diagnosis of type I or type II diabetes or current use of oral blood-sugar-lowering drugs or insulin. PAD was defined according to American Heart Association/American College of Cardiology (AHA/ACC) guidelines [14]. Regular use of antihypertensive medications, antiplatelet drugs, statins or other hypolipidemic drugs and hypoglicaemics was also recorded on a standardized form.

A single neurologist evaluated cognitive performance and asked patients to complete questionnaires: Patient Health Questionnaire - 9 (PHQ-9) and Medical Outcome Survey Short Form 36 version 2 (SF-36v2) in the presence of an investigator; she was blinded to the basic characteristics of patient data.

### 2.3 Assessment of Cognitive Performance

As Montreal Cognitive Assessment Scale (MoCA) has been approved as a valid screening tool for vascular cognitive impairment [18-20], cognitive testing was performed using Latvian or Russian MoCA version, according to the native language of the participant and instructions given by the authors [21]. It is a 10-minute cognitive screening tool used to detect mild cognitive impairment. The MoCA scores range from 0-30 and are divided into 7 subscores: visuospatial/executive (alternating trail-making, cube copy, clock drawing), naming (lion, rhinoceros, camel), attention (forward and backward digit span, taping to the letter A, subtracting 7s from 100), language (sentence repetition, letter fluency), abstraction (similarities between train and bicycle, watch and ruler), memory (delayed verbal recall of 5 words), orientation (to time and space) and an additional point is given to each patient who has educational experience of 12 years or fewer. A final total score of 26 and above is considered normal [22].

### 2.4 Assessment of Depressive Symptoms

PHQ-9 is a well-validated measure that can establish a provisional depressive disorder diagnosis [23]. The PHQ-9 score ranges from 0 to 27 because each of the 9 items can be scored from 0 (“not at all”) to 3 (“nearly every day”). Cut-points 5, 10, 15 and 20 represent the threshold for mild, moderate, moderately severe and severe depression, respectively. PHQ-9 score of 10 or greater, which has sensitivity for major depression of 88%, a specificity of 88%, is recommended to use as a screening cut-point [24]. Therefore, Latvian and Russian versions of the Patient Health Questionnaire (PHQ-9) depression scale [25] was used to assess depressive symptoms. Patients were categorized in two groups according to PHQ-9 score. PHQ-9 scores lower than 10 denoted no relevant depressive symptoms. Scores of 10 or higher indicated relevant depressive symptoms.

### 2.5 Assessment of Health Related Quality of Life

HRQoL was assessed using the Medical Outcome Survey Short Form 36 version 2 (SF-36v2), Latvian and Russian language versions [26].The SF-36v2 includes one favourably scored scale measuring each of eight health domains: physical functioning, role participation with physical health problems (role-physical), bodily pain, general health, vitality, social functioning, role participation with emotional health problems (role-emotional) and mental health. For each item, scores are coded, summed and transformed into a scale from 0 (worst possible health state measured by the questionnaire) to 100 (best possible health state). A difference of 5 to 10 points is considered a clinically important change for an individual subject (a smaller difference may be important for group comparisons) [27]. In addition, the SF-36v2 provides summary scales for overall physical and mental health, which are standardized to a population mean of 50 and a standard deviation of 10 and for which individual differences of 2,5 to 5 points are considered clinically meaningful [28]. SF-36 data of age-matched general Latvian population (age ≥ 66 years) [29] were used to compare HRQoL of general population with severe CAS and PAD.

### 2.6 Ethics Approval and Consent to Participate

The study protocol was approved by the corresponding local ethic committee of Riga Stradins University. Written informed consent was obtained from all patients prior to study inclusion.

### 2.7 Statistical Analysis

Descriptive statistics were used to analyse the demographics and clinical characteristics of the population. Continuous variables were described as means (standard deviation (SD)) or median and interquartile range ([IQR]). Categorical variables were presented as counts and percentages.

The normal distribution of data was tested with the Kolmogorov-Smirnov test. Normally distributed data were analysed with *t* tests for independent samples. Continuous (non-Gaussian distribution) variables were compared with the Mann-Whitney *U* test. To explore comparability of both groups (whether there are any potential cofounders that could influence the results) univariate analysis was carried out for an association between both groups (Chi-square test for categorical variables). A two-sided *p*-value < 0.05 was considered statistically significant. To understand whether differences were statistically meaningful, Cramer's V (<0.3=small effect size, 0.3 - 0.5 = medium effect size, >0.5=large effect size) for Pearson Chi-square test, Cochen's *d* value (<0.5=small effect size, 0.5-0.8=medium effect size, >0.8=large effect size)for *t*-test and *r* (<0.3=small effect size, 0.3-0.5=medium effect size, >0.5=large effect size) for Mann-Whitney was used. Statistical analyses were performed using IBM SPSS Statistics(version 23 for Windows, IBM Corp., Somers, NY, USA).

## 3 RESULTS

### 3.1 Characteristics of Patients

The study comprised of 55 patients with severe CAS and 54 matched control subjects with severe PAD. CAS and control subjects were balanced in regard to age, gender, BMI, and co-morbidities such as coronary artery disease, chronic heart failure, atrial fibrillation and diabetes mellitus. Statistically significant differences were found across groups for TIA or minor stroke, AH, smoking habit and use of medications, including antiplatelet and hypolipidemic drugs. Diagnosis of AH seemed to be more prevalent in patients with severe CAS (*p*=0.001; Carmer's V = 0.367) but compliance of AH treatment and maintenance of normal arterial blood pressure were similar in both groups (*p*=0.951; Cramer's V=0.146). Control subjects with severe PAD presented a higher prevalence of smoking habit (*p*=0.02, Cramer's V = 0,2), and lower compliance of medication treatment (*p*<0.05; Cramer's V= 0.2) although effect size of statistically significant difference was small (Table **1**).

CAS, carotid artery stenosis; PAD, lower extremity peripheral artery disease; SD, standard deviation; TIA, transient ischemic attack; AH, arterial hypertension; NYHA, New York Heart Association classification; BMI, body mass index

Comparison of cognitive performance between patients with carotid artery stenosis and lower extremity peripheral artery disease.

Although both groups presented mean MoCA scores below the suggested cut-off score, mean MoCA scores 24.2 (2.77)were significantly lower in CAS patients than in patients with PAD - 25.8 (2.28) (p<0.01, Cochen's *d* value 0.59).Complementary statistically significant difference with medium effect size was observed with median MoCA scores (*p*=0.005; effect size *r*=0.3) Table (**2**). In our study, patients with CAS performed significantly worse on the MoCA subtests of delayed recall (*p*=0.023, *r*=0.24).

CAS, carotid artery stenosis; PAD, lower extremity peripheral artery disease; MoCA, Montreal Cognitive Assessment Scale; VSE, visuospatial/executive functions

Comparison of depressive symptoms between patients with carotid artery stenosis and lower extremity peripheral artery disease.

According to the accepted threshold of the PHQ-9 (score≥10) for depressive symptoms, the difference of frequency was not statistically significant between both groups Table (**3**). Further, there was no statistically significant difference of median PHQ-9 scores in the CAS group (median PHQ-9 score 4.0 [5]) and in the PAD group (median PHQ-9 score 5.5 [7]), (*p*=0.08, effect size *r*=0.18).

Comparison of HRQoL between patients with carotid artery stenosis and lower extremity peripheral artery disease.

Mean SF-36v2 scores for bodily pain and vitality were significantly lower in patients with severe PAD than in patients with severe CAS (*p*=0.001 and *p*=0.02). The lowest SF-36v2 mean scores in patients with CAS were for general health and the highest for social functioning and mental health. The lowest scores in patients with severe PAD were for physical functioning, role-physical, bodily pain and general health, but the highest was for social functioning and role-emotional. Comparing mean SF-36v2 scores of patients with CAS with those of the general Latvian population aged ≥66 years, there was no statistically significant difference observed. Mean SF-36v2 scores for physical functioning, role-physical and bodily pain were significantly lower in patients with PAD than in the Latvian population under age 66 years (Cohen's *d* value 0.7) (Table **4**).

In mean SF-36v2 scores related to gender and age, there were no statistically significant differences in the CAS or PAD groups. In analysis of whether cognitive impairment (MoCA< 26) had an association with HRQoL either in patients with CAS or PAD, no significant differences were noted between groups. An association between depressive symptoms and impaired HRQoL was observed in both groups. Mean SF-36v2 scores for bodily pain, general health, vitality, social functioning, role-emotional and mental health were significantly lower in patients with depressive symptoms in CAS group Table (**5**). Mean SF-36v2 scores for general health, vitality, role-emotional and mental health were significantly lower in patients with depressive symptoms in the severe PAD group, but there was no significant difference of mean SF-36v2 scores for role-physical and social functioning regardless of whether the patient had depressive symptoms (Table **6**).

## 4 DISCUSSION

Severe CAS is associated with increased risk for cognitive impairment [7], [30] despite the fact that some studies have failed to identify cognitive abnormalities in patients with this condition [31]. It is considered that cognitive impairment in patients with symptomatic severe CAS is in the context of the acute stroke lesion [32, 33], but association of asymptomatic severe CAS and cognitive impairment is not yet clear. One probable pathogenetic mechanism of cognitive impairment in asymptomatic severe CAS may be due to decreased cerebral blood flow (hypoperfusion, microemboli, altered cerebrovascular reactivity) [7]. Another assumption of disputable mechanisms of cognitive impairment is whether carotid stenosis is an indicator or marker for underlying multiple vascular risk factors that predispose patients to cognitive impairment due to intracranial atherosclerosis [34, 35]. Therefore, taking these considerations into account, the control group in our study was chosen to present the same vascular risk factors as those in the severe CAS group, with only one difference: in the control group CAS was <50%. Although overall the vascular risk factors were similar between the two groups in our study, the frequency of previous minor stroke or TIA, AH, smoking habits and medication adherence were significantly different (*p*< 0.05). However, the effect size of statistical significance was small for each of these factors, with the exception for AH. We therefore reasoned that these two groups were comparable in both demographics and vascular risk factors.

The current study found that median MoCA scores were lower in patients with CAS than in those in the control group. This suggests that patients with advanced atherosclerosis and severe CAS may be at higher risk for cognitive impairment than patients with advanced atherosclerosis without CAS. However, because of the known associations of cognitive impairment with minor stroke and TIA, our data should be interpreted with caution, despite the fact that in severe CAS group the number of patients with minor stroke or TIA (n=11; 20%) and the corresponding effect size of statistical significance (Cramer's V = 0.285) were small. Several studies have evaluated cognitive impairment after TIA or minor ischemic stroke. The aims of those studies were similar - to evaluate the frequency of cognitive impairment in patients with TIA [36] or in stroke patients with no significant disabilities using either modified Rankin Scale (mRS) [33] or NIHSS [37] for clinical neurological assessment. There were differences between the studies in the sample characteristics, clinical assessment methods used and timing of cognitive testing, as well as in the definition of TIA (clinical versus imaging based diagnosis of TIA). Nevertheless the conclusions of the studies were consistent: cognitive impairment was common in patients who had suffered an ischemic stroke and had a successful clinical recovery with no functional disability. Additionally, at least one-third of TIA patients had impairment of one or more cognitive domains [38]. However there is still lack of comparable studies and available data on risk factors or potential causes and underlying mechanisms of cognitive dysfunction after TIA. Another reason to interpret the results of the current study with caution is the high prevalence of AH in the CAS group. It is known that high blood pressure is a strong risk factor for white matter lesion (WML) [39] and data suggests that WMLs could lead to cognitive decline and may play a role in the aetiology of dementia [40]. However, there are data that implies that AH treatment could reduce WML progression [39]. Besides, it is also known that AH in small portion of patients with bilateral severe CAS may have a adjusting role in hemodynamics of cerebral perfusion [41], suggesting that in the context of CAS, it may be a protective factor against cognitive dysfunction. For these reasons, the influence of AH on cognitive decline in our study remains uncertain. Finally, patients had modifiable risk factors for vascular disease that have been shown to increase the risk for cognitive impairment [34]. Although the prevalence of these risk factors was similar between the experimental and control groups, they may have influenced cognitive performance in our study, thus the sole impact of severe CAS on cognition may be affected or left obscure. Nevertheless, these data could be considered because, besides being the best medical treatment for CAS, revascularisation may not only prevent risk of stroke but also improve [42-44] or provide some protection against cognitive decline in the elderly [31], [45]. When the results of our study are compared with other studies that used MoCA as a screening tool to assess cognitive performance [45], [46], there were some slight discrepancies, which may be due to disparities in the quantification of CAS and calculation of mean MoCA scores. However, the results were consistent that cognitive impairment was present in patients with severe CAS [47]. This finding was confirmed by additional studies using sensitive standardised complex neuropsychological tests [8], [48].

Depressive symptoms are frequently found in older patients with symptomatic severe CAS [49], but it is unknown whether there is a direct causal relationship between severe CAS and depressive symptoms [50] or whether depression is a consequence of the cerebrovascular atherosclerosis sequelae [51-54]. Although the present study did not identify a statistically significant difference in the frequency of relevant depressive symptoms between the CAS and PAD groups, there was a trend towards more depressive symptoms with higher PHQ-9 scores in patients with PAD. By contrast, similar studies have found that relevant depressive symptoms were more common in patients with CAS than in those with PAD [49], [50]. One possible reason for this discrepancy could be the selection bias for the PAD group in our study. The patients in the PAD group had more advanced PAD and experienced symptoms that interfered with their daily activities. This was confirmed by lower mean scores on the HRQoL evaluation for physical functioning, general health and mental health perception (depressive symptoms and hopeless feeling) for the PAD patients compared to the CAS patients. This is consistent with recently published data, which found that patients with increased atherosclerosis and diminished walking function, were at increased risk for depression [55]. In our study, patients taking antidepressant therapy were excluded because evidence suggests that major depressive disorder and active pharmacological therapy both may affect cognitive function [56].

To further evaluate HRQoL, patients with severe CAS and those with PAD were compared to healthy age matched Latvian population. Evaluating HRQoL, there was no significant difference between the Latvian population under 66 years of age and patients with severe CAS. By contrast, patients with PAD had significantly lower mean SF-36v2 scores for physical functioning, role-physical and bodily pain than did the Latvian population and significantly lower scores for bodily pain and vitality than did patients in the CAS group. There were no associations of probable impact of age, gender or cognitive impairment on HRQoL scores in the CAS and PAD groups. The analysis of HRQoL in patients with and without depressive symptoms in each group showed that the mean SF-36v2 scores were lower for general health, vitality, role-emotional and mental health for patients with depressive symptoms in CAS and PAD groups compared to those without depressive symptoms. These results demonstrate that depression, which has a higher incidence in patients with cardiovascular diseases [57], could lead to a poorer HRQoL. Comparing mean SF-36v2 scores in patients with depressive symptoms, lower scores for physical functioning, bodily pain, general health and mental health were achieved by patients in the PAD group than by patients in the CAS group. These results could be explained by bodily pain and impairment of physical functioning experienced by patients with severe PAD as they have more physically unpleasant symptoms than patients with severe CAS.

Several studies have assessed HRQoL in patients with CAS and PAD with varying results. Some studies reported poorer HRQoL in patients with CAS than in the general population [58], [59]. This contradicts the results of our study, in which there were no significant differences in mean SF-36v2 scores between the severe CAS group and the age-matched Latvian population. This discrepancy could be explained by differences in HRQoL questionnaires, control groups (general rather than age-matched population) and the presentation of the results (median rather than mean values). The Athero-Express biobank study [58] evaluated HRQoL in patients with PAD compared with healthy Dutch age-matched individuals. The findings from this study were consistent with those from our study, demonstrating poorer HRQoL in patients with PAD. However, the Dutch study did not identify a significant difference in HRQoL between patients with CAS and those with PAD, whereas in our study, scores for bodily pain and vitality were significantly lower in the PAD group. The reason for the discrepancy is likely that in our study, all patients in the severe CAS group also had mild to moderate PAD, whereas in the control group, all patients had significant clinical symptoms of PAD with indications for revascularisation.

The PARTNERS study compared HRQoL among patients with PAD and PAD together with other cardiovascular disease (CVD). This study found that HRQoL was lower in the PAD-other-CVD patient group [60]. These results contradict those of our study which found that patients with severe CAS and mild to moderate lower extremity PAD did not have worse HRQoL compared to those with severe PAD alone. The PARTNERS study [60] included not only patients with stroke or TIA, but also those with symptomatic coronary artery disease in the PAD-other-CVD group. Thus, the underlying lower extremity or cardiac symptoms may have influenced impairment of HRQoL, especially the physical components. There are no physical symptoms associated with CAS that could interfere with daily activities and therefore significantly influence HRQoL.

Our results identified an association between severe CAS and cognitive impairment, and between severe PAD and a tendency towards increased frequency of depressive symptoms and lower HRQoL scores. Health care specialists should be aware of these findings when managing patient care because patients may benefit not only from cardiovascular risk factor management but also from additional physical and emotional support that may help to improve their activities of daily living [58]. Additionally, this information can help in decision-making for revascularisation therapy in patients with CAS.

Several limitations of this study should be acknowledged. First, this was a cross-sectional study with a relatively small sample size and a control group size that did not exceed the case sample. Second, there was a higher prevalence of TIA, minor stroke and AH in the CAS group which may have negatively influenced cognitive performance. Future studies should involve recruitment of more asymptomatic CAS and control patients with a better balance of vascular risk factors. Additionally, brain imaging should be performed in both groups to evaluate the presence of WMLs. Finally, the control group exhibited more severe clinical symptoms of PAD than those in the CAS group, which may have influenced the HRQoL data and the frequency of depressive symptoms. Therefore, further studies are needed to explore the influence of severe CAS on cognitive function and development of depressive symptoms using a healthy, age-matched control group.

## CONCLUSIONS

In summary, our findings indicate that severe CAS could play a role in cognitive decline. Further studies should be conducted using larger patient cohorts without ischemic brain lesions and with balanced vascular risk profiles to investigate impact of CAS on cognition. There was no association between severe CAS and depressive symptoms in the present study. As patients with severe CAS did not exhibit physical symptoms, HRQoL was better for those patients than for patients with lower extremity PAD.

## Figures and Tables

**Table 1:** Demographic data and clinical characteristics of patients with severe CAS and control subjects with severe PAD.

**Variable**	**CAS (n=55)**	**PAD (n=54)**	***p* value**	**Effect size**
**Age,Years (SD)** **(Range of Years)**	68,0 (10,3) (44-86)	64,6 (9.7) (45-87)	0.768	Cochen's *d* = 0.34
**Male**	43 (78%)	40 (74%)	0.615	
**Previous Minor Stroke/TIA**	11 (20%)	0	0.001	Cramer's V= 0.285
**TIA/** ***Amaurosis Fugax***	6	0		
**Minor Stroke**	5	0		
**Coronary Disease**	22 (40%)	15 (28%)	0.443	Cramer's V= 0.566
**AH**	54 (98%)	41 (76%)	0.001	Cramer's V= 0.367
**Compliance of Hypertension Treatment**	28 (52%)	21 (51%)	0.951	Cramer's V= 0.146
**Chronic Heart Failure >2 NYHA**	22 (40%)	15 (28%)	0.178	Cramer's V = 0.120
**Atrial Fibrillation**	8 (14%)	5 (9%)	0.395	Cramer's V= 0.075
**Diabetes Mellitus**	6 (11%)	12 (22%)	0.112	Cramer's V= 0.148
**Smoking Habit**	35 (64%)	45 (83%)	0.02	Cramer's V= 0.221
**BMI**	26.8 (4.07)	25.27 (4.21)	0.093	Cochen's *d*= 0.369
**Use of Antiplatelet Drugs**	39 (71%)	27 (50%)	0.02	Cramer's V= 0.2
**Use of Hypolipidemic Drugs**	36 (65%)	17 (32%)	0.001	Cramer's V= 0.3

Data presented as mean (SD) or *n* (%)

**Table 2:** MoCA scores for patients with severe CAS and for control subjects with severe PAD.

	**CAS** (n=55)			**PAD** (n=54)			***p* value**	**Effect Size, *R***
	**Median**	**Q1**	**Q3**	**Median**	**Q1**	**Q3**		
MoCA Total Score	24	23	26	26	25	28	0.005	0.3
MoCA Subtests:								
VSE	4	3	5	4	4	5	0.128	0.16
Naming	3	3	3	3	3	3	0.419	0.08
Attention	5	5	6	6	5	6	0.142	0.16
Language	2	1	2	2	1	2	0.661	0.05
Abstraction	2	1	2	2	1	2	0.773	0.03
Delayed Recall	2	1	4	3.5	2	5	0.023	0.24
Orientation	6	6	6	6	6	6	0.684	0.04

CAS, carotid artery stenosis; PAD, lower extremity peripheral artery disease; MoCA, Montreal Cognitive Assessment Scale; VSE, visuospatial/executive functions

**Table 3:** The frequency of PHQ-9 scores in patients with CAS and in patients with PAD.

	**PHQ-9 scores**			***p* Value**	**Cramer's V**
	**1-4**	**5-9**	**≥10**		
**CAS** (n=55)	32 (58%)	15 (27%)	8 (15%)	0.168	0.168
**PAD** (n=54)	24 (44%)	13 (24%)	17 (32%)		

PHQ-9, Patient Health Questionnaire - 9; CAS, carotid artery stenosis; PAD, lower extremity peripheral artery disease;

**Table 4:** Mean SF-36v2 scores in patients with severe CAS, severe PAD and in the Latvian population ≥ 66 years sample.

**SF-36v2 Subscales**	**CAS** **(n=55)**	**PAD (n=54)**	**Latvian Population ≥ 66 Years** **(n=119)**	**Mean SF-36v2** CAS *vs.* PAD*	**Mean SF-36v2** **CAS *vs.* Latvian Population ≥ 66 Years^#^**	**Mean SF-36v2** **PAD *vs.* Latvian Population ≥ 66 Years^^^**
**PF**	55.3 (23.7)	46.4 (21.6)	63.4(25.5)	0.39	0.33	0.7
**RP**	51.5 (25.6)	42.8 (25.8)	60.9(26.4)	0.34	0.36	0.69
**BP**	55.8 (25.7)	37.7 (20.9)	55.5(24.7)	0.77	<0.1	0.78
**GH**	48.4 (12.0)	43.4 (15.3)	43.7(19.5)	0.36	0.29	<0.1
**VT**	60.6 (15.6)	51.4 (21.2)	55.4(18.4)	0.49	0.3	0.2
**SF**	68.6 (25.4)	59.8 (31.4)	70.5(24.6)	0.31	<0.1	0.38
**RE**	64.2 (25.2)	59.9 (29.1)	70.4(25.9)	0.16	0.24	0.38
**MH**	68.5 (14.8)	61.8 (20.2)	61.1(17.8)	0.38	<0.1	0.45

SD, standard deviation; CAS, carotid artery stenosis; PAD, lower extremity peripheral artery disease; PF - Physical functioning; RP - role physical; BP - bodily pain; GH - general health; VT - vitality; SF - social functioning; RE - role emotional; MH - mental health * Comparing mean SF36v2 between CAS un PAD groups (Cohen's *d* value); **^#^**comparing mean SF36v2 between CAS and Latvian age matched population (Cohen's *d* value); **^^^**comparing mean SF36v2 between PAD and Latvian age matched population (Cohen's *d* value).

**Table 5:** Mean SF-36v2 scores in patients with severe CAS with and without depressive symptoms.

	**CAS Patients, SF-36v2 Mean Scores, (SD)**			
	**PHQ-9 <10** (n=48)	**PHQ-9 ≥10** (n=7)	*P* Value	Cohen's *d* Value
**PF**	56,4 (24.5)	48.8 (18.1)	0.4	0.35
**RP**	53.6 (26.1)	39.1 (19.7)	0.14	0.63
**BP**	59.1 (25.9)	36.4 (12.7)	0.019	1.11
**GH**	49.9 (11.6)	39.4 (10.5)	0.02	0.95
**VT**	64.6 (12.7)	36.7 (7.0)	<0.001	2.72
**SF**	73.1 (24.3)	42.2 (13.2)	0.001	1.58
**RE**	67.4 (25.8)	45.8 (8.9)	0.024	1.12
**MH**	71.1 (14.1)	53.1 (9.2)	0.001	1.51

CAS, severe carotid artery stenosis; PHQ-9, Patient Health Questionnaire - 9; PF - Physical functioning; RP - role physical; BP - bodily pain; GH - general health; VT - vitality; SF - social functioning; RE - role emotional; MH - mental health

**Table 6:** Mean SF-36v2 scores in patients with severe PAD with and without depressive symptoms.

	**PAD Patients, SF-36v2 Mean Scores, (SD)**			
	**PHQ-9 <10** (n=22)	**PHQ-9 ≥10** (n=10)	*P* value	Cohen's *d* Value
**PF**	50.2 (24.1)	38.0 (3.7)	0.14	0.71
**RP**	46.0 (26.7)	35.6 (23.6)	0.299	0.41
**BP**	41.5 (22.3)	29.3 (15.3)	0.13	0.64
**GH**	48.7 (13.1)	31.7 (13.5)	0.002	1.28
**VT**	57.4 (20.6)	38.1 (16.3)	0.014	1.04
**SF**	63.1 (33.1)	52.5 (27.5)	0.386	0.35
**RE**	66.7 (30.1)	45.0 (21.2)	0.049	0.83
**MH**	69.5 (18.1)	44.5 (12.8)	<0.0001	1.60

CAS, severe carotid artery stenosis; PHQ-9, Patient Health Questionnaire - 9; PF - Physical functioning; RP - role physical; BP - bodily pain; GH - general health; VT - vitality; SF - social functioning; RE - role emotional; MH - mental health

## LIST OF ABBREVATIONS

HRQoL: = Health related quality of life;
CAS: = Carotid artery stenosis;
PAD: = Lower extremity peripheral artery disease
MoCA: = Montreal Cognitive Assessment
PHQ-9: = Patient Health Questionnaire – 9
SF-36v2: = Medical Outcome Survey Short Form version 2
ACD: = Atherosclerotic cardiovascular disease
CAE: = Carotid artery endarterectomy;
NASCET: = North American Symptomatic Carotid Endarterectomy Trial;
NIHSS: = National Institute of Health Stroke Scale
TIA: = Transient ischemic attack
ACAS: = Asymptomatic Carotid Atherosclerosis Study
BMI: = Body mass index
NYHA: = New York Heart Association classification
AHA/ACC: = American Heart Association/ American College of Cardiology Foundation
SD: = Standard deviation
IQR: = Interquartile range
VSE: = Visuospatial/executive functions
PF: = Physical functioning
RP: = Role physical
BP: = Bodily pain
GH: = General health
VT: = Vitality
SF: = Social functioning
RE: = Role emotional
MH: = Mental health
PARTNERS: = Peripheral Arterial Disease Awareness, Risk and Treatment: New Resources of Survival Program
CVD: = Cardiovascular disease
mRS: = Modified Rankin Scale
AH: = Arterial hypertension
WML: = White matter lesion
